# Seasonal Variation and Resin Composition in the Andean Tree *Austrocedrus chilensis*

**DOI:** 10.3390/molecules19056489

**Published:** 2014-05-21

**Authors:** Verónica Rachel Olate, Alex Soto, Guillermo Schmeda-Hirschmann

**Affiliations:** 1 Instituto de Química de Recursos Naturales, Laboratorio de Química de Productos Naturales, Universidad de Talca, Casilla 747, 3460000 Talca, Chile; 2 Instituto de Matemática y Física, Universidad de Talca, Casilla 747, 3460000 Talca, Chile

**Keywords:** *Austrocedrus chilensis*, Cupressaceae, diterpenes, principal component analysis, seasonal composition, resin

## Abstract

Little is known about the changes in resin composition in South American gymnosperms associated with the different seasons of the year. The diterpene composition of 44 resin samples from seven *Austrocedrus chilensis* (Cupressaceae) trees, including male and female individuals, was investigated in three different seasons of the year (February, June and November). Twelve main diterpenes were isolated by chromatographic means and identified by gas chromatography-mass spectrometry and nuclear magnetic resonance (NMR). The diterpene composition was submitted to multivariate analysis to find possible associations between chemical composition and season of the year. The principal component analysis showed a clear relation between diterpene composition and season. The most characteristic compounds in resins collected in summer were *Z*-communic acid (**9**) and 12-oxo-labda-8(17),13*E*-dien-19 oic acid methyl ester (**10**) for male trees and 8(17),12,14-labdatriene (**7**) for female trees. For the winter samples, a clear correlation of female trees with torulosic acid (**6**) was observed. In spring, *E*-communic acid (**8**) and *Z*-communic acid (**9**) were correlated with female trees and 18-hydroxy isopimar-15-ene (**1**) with male tree resin. A comparison between percent diterpene composition and collection time showed *p* < 0.05 for isopimara-8(9),15-diene (**2**), sandaracopimaric acid (**4**), compound (**7**) and ferruginol (**11**).

## 1. Introduction

The dioecious tree *Austrocedrus chilensis* (D. Don) Florin et Boutelje (Syn.: *Austrocedrus chilensis* (D. Don) Pic. Ser. et Bizz, Cupressaceae) is a characteristic gymnosperm of the southern Andes slopes and is locally known as “ciprés de cordillera”. The distribution range of *A. chilensis* covers two wide parallel areas in the Cordillera de los Andes, in both sides of the mountain range in Chile and Argentina. It includes the west Andean populations in the Mediterranean region of Chile, east Andean populations ranging from humid rain forest to the steppe ecotone and coastal mountain populations from Mediterranean Chile [1,2,3]. This species is more common in the eastern Andes (Argentina), where the wide precipitation gradient ranges from 2500 to 4000 mm per year [3]. The Cupressaceae *Austrocedrus chilensis* covers some 47,000 ha of native forest in Chile [4]. Previous studies on the chemical composition of this species have been centered mainly in its wood and volatile compounds [5,6]. A recent investigation reported the resin composition of *A. chilensis* from the western Andean slopes as a complex mixture of diterpenes, with 17 compounds being identified, 10 of them as main constituents [7]. When single drops of resin naturally exuded from the trees in different seasons were separately analyzed by gas chromatography-mass spectrometry (GC-MS) and proton nuclear magnetic resonance (^1^H-NMR), qualitative and quantitative differences in the main constituents were observed. To confirm or discard the hypothesis that the composition of the resin diterpenes can be associated with the season of the year, a study was undertaken in a mature tree population to compare the composition of freshly exuded drops of resin of individual female and male trees collected in spring, summer and winter. The results were compared by multivariate statistical methods (Principal Component Analysis, PCA).

Multivariate statistical analysis is used as a valuable tool for biometric analysis of plant populations [8,9,10]. It is extensively employed to detect differences in chemical composition that can be associated to parameters such as age, sex and mating status [11] to discriminate between sample origin in medicinal and food plants [12] and archaeological samples [13]. Different methods are used to obtain information on chemical constituents of the samples to be analyzed by PCA, including matrix-assisted laser desorption-mass spectrometry (MALDI-MS) [11], surface desorption atmospheric pressure chemical ionization mass spectrometry (DAPCI-MS) [12] and nuclear magnetic resonance (NMR) [14,15,16,17,18]. The terpene composition of gymnosperms has been used to assess the genetic diversity of *Pinus* and *Thuja* species [19,20]. The aim of our study was to determine possible differences in the resin composition of *A. chilensis*, a dioecious tree, according to the season. Resins from female and male trees were individually collected and analyzed to find possible differences according to the season of the year.

## 2. Results and Discussion

**Figure 1:**
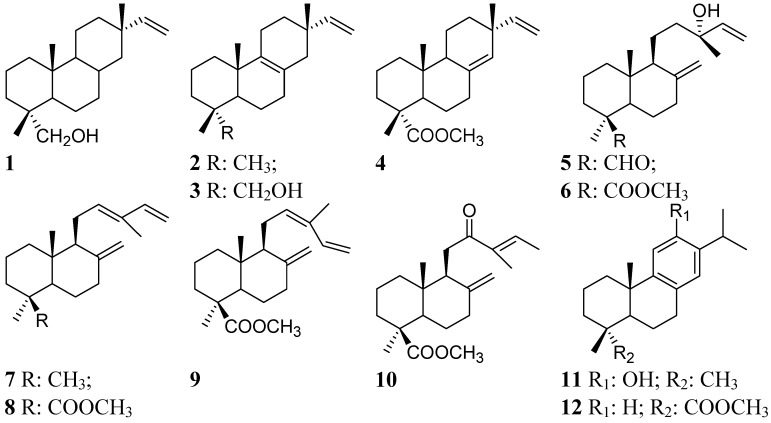
Structure of the main compounds from the resin of *Austrocedrus chilensis* used for the PCA analysis. Compounds: 18-hydroxyisopimar-15-ene (**1**); isopimara-8(9),15-diene (**2**); 18-hydroxyisopimara-8,15 diene (**3**); sandaracopimaric acid methyl ester (**4**); torulosal (**5**); torulosic acid methyl ester (**6**); 8(17),12,14-labdatriene (**7**); *E*-communic acid acid methyl ester (**8**); *Z*-communic acid methyl ester (**9**); 12-oxo-labda-8(17),13*E*-dien-19-oic acid methyl ester (**10**); ferruginol (**11**); dehydroabietic acid methyl ester (**12**).

**Figure 2:**
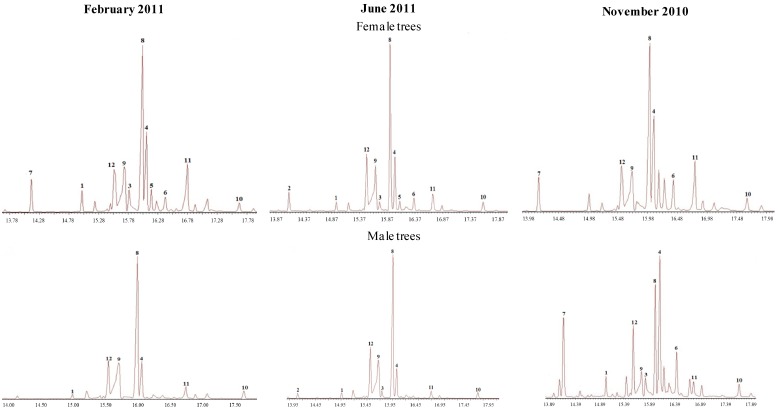
Gas chromatography traces of female and male *Austrocedrus chilensis* resin samples collected in different seasons of the year (as methyl esters). Compounds were identified by comparison with standards isolated from the resin and comparison of the mass fragmentation patterns with literature. Compounds: **1**: 18-hydroxy isopimar-15-ene; **3**: isopimara-8(9), 15-dien-19-ol; **4**: sandaracopimaric acid methyl ester; **5**: torulosal; **6**: torulosic acid methyl ester; **7**: 8(17),12,14-labdatriene; **8**: *E*-communic acid acid methyl ester; **9**: *Z*-communic acid methyl ester; **10**: 12-oxo-labda-8(17),13*E*-dien-19 oic acid methyl ester; **11**: ferruginol; **12**: dehydroabietic acid methyl ester.

The main 12 diterpenes isolated/identified from the resin of *Austrocedrus chilensis* were used for PCA analysis. The structure and identity of the compounds are presented in Figure 1 and the origin of the resin samples is summarized in Table 1. Representative GC chromatograms of the resins are shown in Figure 2. The relative percent composition of the diterpenes used for PCA analysis in the February, June and November samples is shown in Table 2, Table 3, and Table 4. Additional information regarding to the contribution of the new components to the total variance, the comparison between diterpene composition, tree sex (female or male) and collection time (month/season), the single compound contribution to the new components according to collection time and single sample contribution to the new components is presenting as Supplementary information (Tables S1-S5). To explain the relation of the *p* = 12 original compounds in *r* *<* *p* components, three new components were selected for the February and June samples, explaining 80.14% and 81.37% of the variability, respectively. For the November samples, four new components were selected, accounting for 86.05% of the variability (Table S1).

**Table 1:** Resin samples collected from *Austrocedrus chilensis* trees according to plant sex and seasons. February, *n* = 15; June, *n* = 14; November, *n* = 15. NC: not collected.

Tree	Sex	Sample number		
		February 2011	June 2011	November 2010
1	Female	101, 102, 103, 104	201, 202, 203	01, 02, 03, 04
2	Male	105, 106	204, 205	NC
3	Female	109, 110	209	05, 06, 07
4	Female	111, 112	210	08, 09, 10
5	Male	113, 114	211, 212	11, 12
6	Male	115	213, 214	13, 14, 15
7	Male	107, 108	206, 207, 208	NC

**Table 2:** Relative percent composition of the *Austrocedrus chilensis* resin diterpenes measured by GC-MS (as methyl esters). Samples collected in February 2011. Samples 101–104 and 109–112 are from female trees, 105–108 and 113–115 from male trees. Nd: not detected.

	Compound number											
	1	2	3	4	5	6	7	8	9	10	11	12
Sex												
Female												
101	2.8	Nd	4.1	9.8	3.3	6.2	7.3	27.8	9.9	Nd	9.5	9.2
102	3.3	Nd	3.3	10.8	2.5	2.7	4.9	35.7	11.6	1.9	10.8	7.8
103	1.9	Nd	Nd	10.9	Nd	Nd	Nd	42.3	20.0	Nd	10.0	12.1
104	2.7	Nd	1.7	13.5	1.2	2.5	3.3	39.6	12.4	1.9	10.0	7.5
109	6.3	Nd	7.8	8.4	4.5	Nd	29.5	17.6	7.2	Nd	3.4	8.8
110	3.9	Nd	2.2	14.0	Nd	1.1	1.4	32.5	17.2	2.4	3.9	12.5
111	5.9	Nd	3.7	8.5	5.0	5.5	13.3	23.8	9.1	1.2	3.7	10.5
112	4.7	Nd	5.5	9.1	3.8	5.0	14.1	22.3	10.4	1.0	3.1	9.3
Male												
105	1.2	Nd	Nd	7.8	Nd	Nd	Nd	45.0	23.4	2.5	3.9	11.9
106	Nd	Nd	Nd	6.6	Nd	Nd	Nd	44.5	26.2	3.6	4.3	12.7
107	4.2	Nd	Nd	10.4	1.9	3.3	21.5	24.5	12.4	1.8	5.2	9.3
108	3.8	Nd	Nd	10.4	Nd	4.4	17.2	23.6	11.9	1.9	4.8	8.9
113	6.2	Nd	6.5	11.5	Nd	Nd	Nd	31.5	12.5	Nd	5.8	9.5
114	8.5	Nd	7.9	10.6	Nd	Nd	Nd	28.9	8.9	Nd	5.9	10.9
115	0.9	Nd	Nd	9.6	Nd	Nd	Nd	33.1	30.2	2.9	4.5	14.2

**Table 3:** Relative percent composition of the *Austrocedrus chilensis* resin diterpenes measured by GC-MS (as methyl esters). Samples collected in June 2011. Samples 201–203 and 209–210 are from female trees, 204–208 and 211–214 from male trees. Nd: not detected.

Sex	Compound number											
	1	2	3	4	5	6	7	8	9	10	11	12
Female												
201	2.4	5.9	2.1	12.3	1.8	3.1	Nd	30.2	14.9	2.9	4.5	15.9
202	2.5	8.5	2.6	13.9	1.5	4.3	Nd	24.4	14.8	2.4	5.6	16.7
203	1.7	4.2	1.8	11.0	1.6	2.4	Nd	34.1	18.6	2.4	4.4	14.9
209	3.8	27.8	7.2	7.3	2.7	2.1	Nd	16.9	9.1	0.3	2.7	7.8
210	2.7	3.3	1.4	12.6	1.3	2.9	Nd	32.3	15.3	2.6	3.4	14.9
Male												
204	Nd	1.7	Nd	12.1	Nd	Nd	Nd	42.8	23.5	1.9	1.4	14.2
205	1.6	1.5	0.7	9.4	Nd	Nd	Nd	48.4	17.4	2.0	2.5	12.9
206	2.7	27.3	2.7	8.6	0.9	Nd	Nd	21.2	11.6	1.9	2.9	9.0
207	3.2	31.8	3.3	6.4	2.9	Nd	Nd	17.9	11.0	0.0	2.9	8.7
208	2.4	23.7	3.2	8.4	1.5	1.7	Nd	21.5	11.1	0.3	3.9	8.3
211	5.8	1.0	8.3	15.2	Nd	Nd	Nd	24.6	9.5	1.0	4.3	17.3
212	Nd	Nd	12.6	10.3	Nd	Nd	Nd	25.8	9.2	1.1	4.1	13.8
213	2.6	4.8	2.0	13.7	1.5	1.7	Nd	39.9	14.4	0.6	2.7	10.8
214	2.6	3.1	3.2	12.9	1.5	2.4	Nd	36.8	15.1	2.8	3.1	11.2

**Table 4:** Relative percent composition of the *Austrocedrus chilensis* resin diterpenes measured by GC-MS (as methyl esters). Samples collected in November 2010. Samples 1–10 are from female trees, 11–15 from male trees. Nd: not detected.

Sex	Compound number											
	1	2	3	4	5	6	7	8	9	10	11	12
Female												
01	Nd	Nd	Nd	4.0	Nd	1.0	0.1	85.0	3.2	0.7	2.7	3.0
02	2.4	Nd	Nd	22.5	Nd	Nd	Nd	35.5	11.4	1.9	17.3	Nd
03	Nd	4.2	Nd	17.6	Nd	4.7	Nd	28.3	11.9	3.0	4.9	12.0
04	Nd	4.1	Nd	8.8	Nd	Nd	Nd	51.7	12.1	1.1	9.7	11.6
05	1.7	Nd	Nd	20.9	Nd	Nd	Nd	36.4	16.5	1.9	8.0	10.3
06	3.5	Nd	Nd	19.4	Nd	Nd	Nd	38.7	18.6	1.8	5.1	11.2
07	Nd	Nd	Nd	25.9	Nd	Nd	Nd	31.6	23.4	2.1	3.3	13.7
08	4.9	Nd	Nd	10.3	3.2	4.5	19.8	20.7	11.0	Nd	2.3	11.4
09	5.3	Nd	7.7	12.8	4.8	11.4	21.2	13.6	4.9	2.1	3.0	13.7
10	Nd	7.5	5.5	34.3	Nd	7.8	Nd	6.8	0.1	9.1	7.2	17.7
Male												
11	2.8	Nd	5.6	16.9	Nd	3.2	Nd	31.5	8.4	Nd	2.0	14.4
12	8.1	Nd	15.4	9.4	Nd	Nd	Nd	12.7	6.0	Nd	20.4	5.9
13	3.5	Nd	3.1	27.3	Nd	7.9	14.1	17.9	3.9	2.8	3.0	10.8
14	4.7	Nd	3.8	17.1	4.0	3.4	11.0	24.7	6.7	2.0	8.2	7.8
15	5.2	Nd	Nd	16.2	4.2	3.1	14.4	31.0	10.1	1.2	3.9	10.8

### 2.1. Percent Diterpene Composition According to Collection Time (Month) and Tree Sex

**Figure 3:**
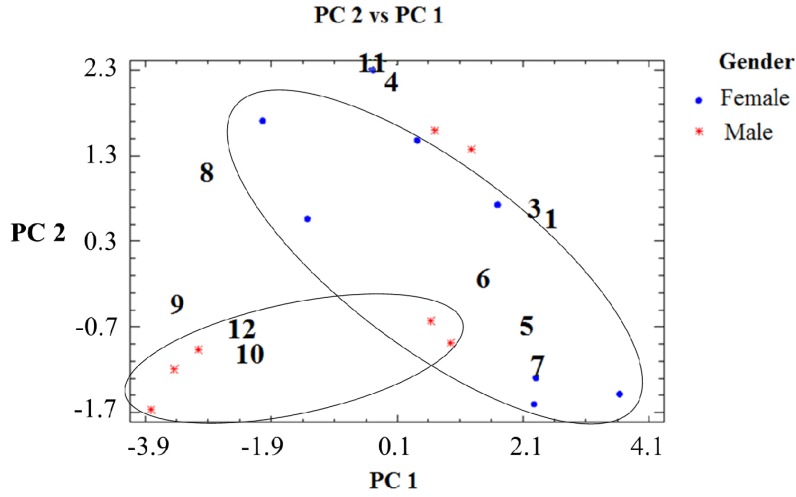
Graphic display of variables for selected components in the February collection.

**Figure 4:**
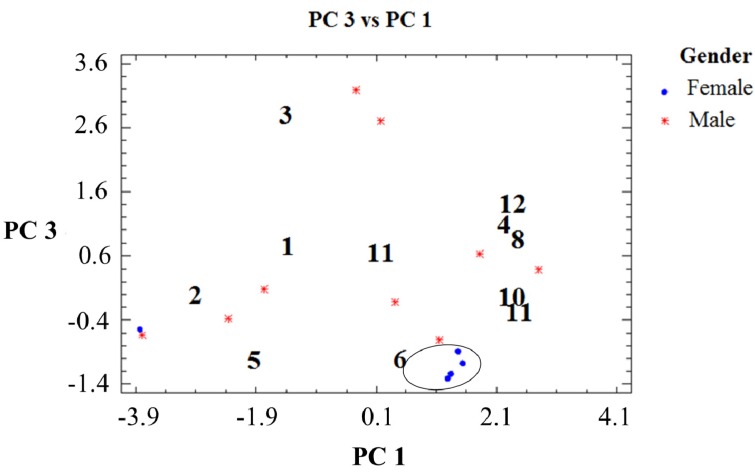
Graphic display of variables for selected components in the June collection.

**Figure 5:**
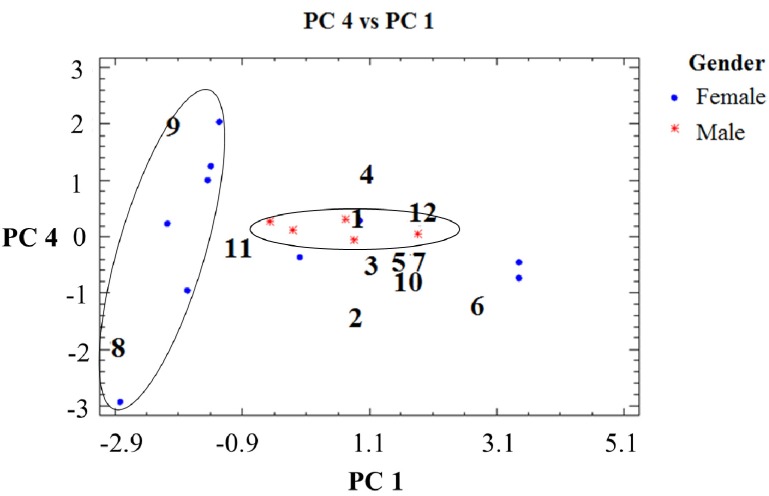
Graphic display of variables for selected components in the November collection.

Graphical display of variables for selected components in the February, June and November collections are presented in Figure 3, Figure 4and Figure 5. The single compound contribution to the new components according to season/month of collection is shown in Table S4 and the single sample contribution to the new components is presented in Table S5. PCA analysis detected differences in resin composition related to collection time and tree sex. The results suggest a differentiation associated with collection month/season and sex. This observation was correlated with the results obtained with the W Mann-Whitney test, which showed significant differences for compounds **1**, **5** and **6** (Table S2). The compound 7 occurs in seven out of eight female samples and only in two out of seven male tree resin samples collected in February (Table 2). Torulosic acid **6** occurred in all female tree resins collected in June (Table 3). In the November samples, the isopimarane **1** was identified in all male resins (Table 4). Torulosal 5 was found in all female samples collected in June (Table 3) and in six out of eight female resins from February (Table 2). The compounds **9**, **10** and **12** could be related with male trees in the February samples (graphic PC2 *vs.* PC1, Figure 3). For the June resins a strong cluster in samples from female trees can be observed in Figure 4 with a clear correlation with compound **6**. The compound **6** occurs in all female trees, but not in all male trees (Table 3, Table S2, *p* = 0.004). Resin samples from male trees are not showing clusters or differences in June. The most relevant graphic display of variables for the November samples is presented in Figure 5, where a strong relation between female tree samples and compounds **8** and **9** can be observed. The Figure 5 also shows a cluster in resin samples from male trees with a close relation with compound **1**. The compound **1** occurs in all male resin samples but only in five out of ten female trees (Table 4). Compound **11** is also related with male trees as well as compound **3**. Compound **3** was found in four out of five male resin samples and only in two out of ten female resins (Table 4). This observation support the assumption that compound **3** is associated to male resins (Figure 5).

### 2.2. Percent Diterpene Composition and Collection Time (Month/Season)

The comparison was performed using the Kruskal-Wallis-test. First, data of all rows were combined and ordered from lower to higher values to calculate the rank for the data of all collections (Table S3). Statistically significant differences between mean values were observed for compounds **2**, **4**, **7** and **11**. Compounds **2** and **4** are pimarane diterpenes, while **7** is a labdane and **11** the abietane ferruginol. Isopimara 8(9),15-diene (2) occurs mainly in the winter resin samples from the southern hemisphere. The labdane **7** was not detected in the June (winter) but in the late spring and summer samples collected in November and February, respectively.

The differences in female and male individuals in dioecious species has been the subject of investigations including variables such as growth, gender, temperature and precipitations [21]. Clear differences in growth rate between tree-ring width, sex, mean temperature and rainfall was reported for the Cupressaceae *Juniperus communis* subsp. *communis* [21]. Different sex-related variables were investigated in *Juniperus virginiana* and *Juniperus oxycedrus macrocarpa*. Differences were found in diameter and height associated with sex, with larger and taller male trees and also a higher concentration of secondary metabolites in male individuals [22,23]. Comparatively less has been done on the association of the variables compound identity and sex in gymnosperms. The variability in the terpene composition of the endemic tree *Fitzroya cupressoides* (Mol.) Johnst. (Cupressaceae) known as “alerce” has been investigated to assess possible changes in composition according to geographic distribution. While the study did not find geographic variability, three different sesquiterpene chemotypes were found [24,25].

Some examples of studies dealing with seasonal variation in terpenes include a report on the essential oil of different parts of the tree and resin of *Protium spruceanum* [26], sesquiterpenes and diterpenes from *Pinus pinaster* needles [27] and rubber and resin content in guayule (*Parthenium argentatum*) [28]. Some seasonal differences in the composition were found for the two chemotypes of *P. pinaster* analyzed. For one of the chemotypes, changes were more significant in the neutral components than in the acid compounds while for chemotype 2 the changes were in the neutral compounds and resin acids. Therefore, the changes are associated with chemotypes and should be interpreted with caution within a population of the species.

The present article shows that diterpene patterns can be associated with the variable season/collection time. Recent studies have succeeded in comparing the chemical composition between male and female plants, including the analysis of secondary metabolites and its fluctuation based on changes of the season [29] and sex related differences [30]. The obtained results also suggest that diterpene resin composition should give information about the variable sex in the dioecious tree *A. chilensis*. However, it is necessary to have a larger number of samples and also more tree individuals, including male and female specimens. On the other hand, as several medicinal plants are dioecious, studies on bioactivity and composition should consider the variable sex when looking for active metabolites.

## 3. Experimental

### 3.1. Plant Material

The resin of *A. chilensis* was collected from four male and three female mature trees from a wild growing population at Las Trancas, VIII Region, Chile (36°54′03′′S, 31°32′47′′W). Single trees were marked and photographed in the plot for identification. The resin is either naturally exuded or as a response from injuries. In the present study, only naturally exuded drops of resin were collected. The naturally exuded resin drops are small in size, colorless to pale yellow or light brown, with a pleasant aroma. For comparison, fresh resin drops (total: 44 samples, ranging from 1 to 4 resin drops from each tree) were separately collected in different seasons from the same trees. Voucher herbarium specimens have been deposited at the Herbario de la Universidad de Concepción (CONC 175068 for female trees; CONC 175069 for male trees) and were identified by Dr. Patricio Peñailillo. The single drop resin samples were collected in glass vials and were taken to the laboratory for analysis. The origin of the samples is summarized in Table 1. For each sample, the selected variables included the relative % composition of the main twelve compounds from the resin. The structure of the compounds is shown in Figure 1. The relative abundance was calculated from the intensity of each single compound in the total chromatogram, considering as 100% the contribution of compounds **1**–**12** in the samples. The resin samples were collected in three seasons of the southern hemisphere, in the months of November (spring), February (summer) and June (winter) as we wanted to know if there was a correlation between season, composition and tree sex.

### 3.2. GC-MS and NMR Analysis

For the GC-MS analyses, the single resin samples were dissolved in chloroform. The organic phase was dried over anhydrous sodium sulphate, filtered and taken to dryness. The dry residue was redissolved in diethyl ether and was methylated with diazomethane to obtain the corresponding methyl esters. According to TLC analyses, the samples consisted of diterpene mixtures. The resin diterpenes, identified in a previous work by spectroscopic and spectrometric means [7] were methylated and characterized by GC-MS to serve as standards for comparison. The equipment used was a Perkin Elmer Turbo Mass (Perkin-Elmer Corporation, Norwalk, CT, USA). Compounds were characterized by electron-ionization (EI) mass spectra. Column: fused silica capillary column, SP-2330 (Supelco), 30 m × 0.25 μm. Carrier: He, split 1:50, initial setpoint: 20.0 PSIG. Oven program: total run time: 66 min, initial temperature: 100 °C, initial hold: 1.00 min, Ramp: 10.0 °C/min to 250 °C, hold for 50.00 min. Injection volumen: 1 μL. The compounds were identified by retention time (min), mass fragmentation pattern and comparison with compounds previously isolated from the resin.

NMR experiments were performed on a Bruker Avance 400 NMR spectrometer (Bruker BioSpin GmbH, Rheinstetten, Germany) equipped with a 5 mm inverse detection z-gradient probe. The ^1^H spectra were measured at 400 MHz at room temperature (22–23 °C) using CDCl_3_ as solvent. Chemical shifts are given on the δ scale and were referenced to residual CHCl_3_ at 7.25 ppm.

### 3.3. Description of the Parameters System

In the present work, 44 single drops of resin from four male and three female *A. chilensis* trees were comparatively analyzed. The main compounds considered for this study were identified by co-chromatography with standards previously isolated from the resin. The derivatization used prior to GC-MS analysis (formation of methyl esters of the diterpene acids) was simple, rapid and efficient affording stable compounds and was preferred over other alternatives, such as preparing TMS derivatives [31].

### 3.4. Principal Component Analysis (PCA)

The relative composition/ratio of diterpenes (compounds **1**–**12**, Figure 1) in the samples was assessed by multivariate statistical methods. Principal Component Analysis was carried out to explain the relation of the *p* = 12 original compounds (**1**–**12**) in *r* *<* *p* components. It allows an optimal representation in a smaller dimensional space (*r*) of the multivariate problem (*p*). Thus, PCA is the first step to identify potential latent variables or not observed variables.

The analysis was based on the correlation matrix. For normalization and scaling, each variable was standardized before calculating covariance, subtracting the mean and dividing between its standard deviation. Data were compared by Pearson’s correlations between each variable pairs. The correlation coefficient rank ranges from −1 to +1 and measures the linear relation between compounds. The differences in diterpene composition according to collection time and sex was carried out using median comparison tests (W-Mann-Whitney and Kruskal Wallis test) due to the number of samples. For the comparison using the Kruskal-Wallis test, the null hypothesis that median of the three collection data is the same for all compounds is tested. First, the data of all rows are combined and ranked from lower to higher values. Then, the mean rank for the data of each collection was calculated. The median values of the diterpenes within female and male trees were combined and ordered from lower to higher values. The mean ranks of the two samples were compared with the combined data. Statistical significance was set at 5%. The information is summarized in Table S2 and Table S3. Based on this information, the PCA was carried out. Some of the twelve compounds used for PCA analysis include sandaracopimaric acid (**4**), torulosic acid (**6**), *E*-communic acid (**8**), *Z*-communic acid (**9**), ferruginol (**11**) and dehydroabietic acid (**12**).

## 4. Conclusions

Diterpenes from the resin of *Austrocedrus chilensis* exhibited differences in its composition according to the season (collection time). These differences were established by statistical means, including PCA analysis and mean comparison tests. PCA analysis also showed a variation in the relative resin diterpene composition regarding to the sex of the trees. This variation was verified using mean comparison tests. The W Mann-Whitney test displayed significant differences in the relative composition of resin diterpenes according to the collection time and tree sex for compounds **1**, **5** and **6**. Compound **1** was more related with male trees in November, while compounds **5** and **6** were related to female trees in February and June, respectively. Kruskal-Wallis test showed significant differences in relation to the collection time for compounds **2**, **4**, **7** and **11**. The most marked differences were related with compound **2** (present only in winter collection) and compound **11** (present in spring and summer collection). This work corresponds to the first report about seasonal differentiation related to resin diterpene composition in native South American gymnosperms.

## Supplementary Materials

Supplementary materials can be accessed at: http://www.mdpi.com/1420-3049/19/5/6489/s1.
