# Supplementary material for: Seasonal Variation and Resin Composition in the Andean Tree Austrocedrus chilensis
Source: Molecules. 2014 May 21;19(5):6489–503. doi: 10.3390/molecules19056489 (PMC6271173; doi:10.3390/molecules19056489)
Supplement: Supplementary file 1 [file molecules-19-06489-s001.pdf]

Supplementary Materials

Table S1. Contribution of new components to the total variance. For the February and June collection, contribution of components **1–11**, for the November collection, components **1–12**.

	February, Summer			June, Winter			November, late Spring		
Component	Eigen value	%Variance	Accumulated percent	Eigen value	%Variance	Accumulated percent	Eigen value	%Variance	Accumulated percent
1	5.15876	46.898	46.898	4.50312	40.937	40.937	3.61955	30.163	30.163
2	2.03656	18.514	65.412	2.54244	23.113	64.051	3.3292	27.743	57.906
3	1.61984	14.726	80.138	1.90513	17.319	81.370	2.12531	17.711	75.617
4	0.826696	7.515	87.653	0.861443	7.831	89.201	1.25137	10.428	86.045
5	0.490514	4.459	92.113	0.431115	3.919	93.120	0.737493	6.146	92.191
6	0.376118	3.419	95.532	0.294093	2.674	95.794	0.514756	4.290	96.481
7	0.258066	2.346	97.878	0.189348	1.721	97.515	0.186237	1.552	98.033
8	0.111761	1.016	98.894	0.166572	1.514	99.030	0.11444	0.954	98.986
9	0.0956063	0.869	99.763	0.0655956	0.596	99.626	0.0534756	0.446	99.432
10	0.0253027	0.230	99.993	0.0341867	0.311	99.937	0.0401717	0.335	99.767
11	0.00077145	0.007	100.000	0.00696071	0.063	100.000	0.0222824	0.186	99.952
12	-	-	-	-	-	-	0.00571465	0.048	100.000

Table S2. Comparison between diterpene composition, sex and collection month/season.

	**Collection month and season of the year**		
Compound (p value)	February (Summer)	June (Winter)	November (late Spring)
1	0.685	0.789	0.046
2	-	0.351	0.220
3	0.232	0.505	0.062
4	0.452	0.689	0.854
5	0.020	0.088	0.479
6	0.127	0.004	0.613
7	0.189	-	0.447
8	0.524	0.594	0.198
9	0.132	0.594	0.198
10	0.261	0.124	0.423
11	1.000	0.095	1.000
12	0.165	0.230	0.500

-: not detected.

Table S3. Comparison between percent diterpene composition and collection month (season).

**Compound**	***p* value**
1	0.182
2	0.000
3	0.352
4	0.006
5	0.471
6	0.452
7	0.004
8	0.856
9	0.061
10	0.747
11	0.022
12	0.168

Table S4. Single compound contribution to the new components according to collection month/season.

**Compound**	**February, Summer**			**June, Winter**			**November, late Spring**			
	**Comp 1**	**Comp 2**	**Comp 3**	**Comp 1**	**Comp 2**	**Comp 3**	**Comp 1**	**Comp 2**	**Comp 3**	**Comp 4**
1	0.351	0.104	−0.395	−0.192	0.323	−0.026	0.163	0.454	0.225	0.217
2	-	-	-	-	-	-	0.160	−0.421	0.123	−0.276
3	0.324	0.113	−0.419	−0.422	−0.093	−0.234	0.209	0.173	0.508	−0.036
4	0.029	0.528	0.033	−0.185	0.259	0.520	0.200	−0.359	0.070	0.412
5	0.331	−0.235	0.230	0.347	0.298	0.087	0.274	0.340	−0.240	−0.007
6	0.236	−0.093	0.534	−0.276	0.101	−0.519	0.480	0.006	−0.120	−0.206
7	0.313	−0.344	0.157	0.106	0.361	−0.511	0.333	0.327	−0.275	0.001
8	−0.374	0.234	0.066	0.381	−0.290	0.002	−0.410	−0.012	−0.242	−0.427
9	−0.418	−0.157	−0.045	0.363	−0.289	−0.249	−0.283	−0.065	−0.219	0.648
10	−0.303	−0.274	0.242	0.365	0.107	−0.231	0.277	−0.414	0.108	−0.072
11	−0.019	0.561	0.354	0.036	0.561	−0.047	−0.144	0.061	0.603	0.070
12	−0.317	−0.218	−0.339	0.363	0.311	0.136	0.319	−0.236	−0.207	0.228

Comp: Component.

Table S5. Single sample contribution to the new components. Month and season.

**February, Summer**				**June, Winter**				**November, late Spring**				
Gender	Comp 1	Comp 2	Comp 3	Gender	Comp 1	Comp 2	Comp 3	Gender	Comp 1	Comp 2	Comp 3	Comp 4
Female	1.701	0.726	1.536	Female	1.343	1.438	−1.247	Female	−2.810	0.394	−0.989	−2.944
Female	0.417	1.480	1.483	Female	1.297	2.700	−1.324	Female	−2.080	0.025	1.568	0.228
Female	−2.033	1.704	−0.050	Female	1.539	0.356	−1.082	Female	0.008	−1.920	−0.397	−0.375
Female	−0.277	2.300	1.595	Female	−3.826	0.072	−0.555	Female	−1.764	−1.096	−0.181	−0.959
Female	3.650	−1.492	−1.295	Female	1.466	0.610	−0.905	Female	−1.447	−0.724	−0.188	0.995
Female	−1.317	0.557	−0.811	Male	2.805	−3.078	0.384	Female	−1.388	−0.418	−0.507	1.249
Female	2.288	−1.604	0.429	Male	1.818	−2.239	0.627	Female	−1.251	−1.592	−1.135	2.038
Female	2.310	−1.304	0.209	Male	−1.774	−0.989	0.068	Female	0.948	2.010	−1.592	0.287
Male	−3.063	−0.963	−0.239	Male	−3.800	−0.990	−0.640	Female	3.462	2.185	−0.754	−0.458
Male	−3.818	−1.671	0.060	Male	−2.361	−0.216	−0.380	Female	3.453	−4.341	1.444	−0.747
Male	0.958	−0.886	1.030	Male	0.168	2.453	2.711	Male	−0.101	0.046	−0.171	0.118
Male	0.641	−0.627	1.078	Male	−0.232	0.498	3.189	Male	−0.466	2.405	4.211	0.273
Male	0.707	1.599	−1.823	Male	0.411	−0.663	−0.122	Male	1.864	−0.099	−0.194	0.052
Male	1.285	1.380	−2.667	Male	1.146	0.048	−0.723	Male	0.850	1.528	0.129	−0.061
Male	−3.448	−1.198	−0.534	-	-	-	-	Male	0.722	1.596	−1.244	0.305

Comp: Component.
